# Understanding the Clinical Implications of Intracranial Arterial Calcification Using Brain CT and Vessel Wall Imaging

**DOI:** 10.3389/fneur.2021.619233

**Published:** 2021-07-15

**Authors:** Wen-Jie Yang, Bruce A. Wasserman, Lu Zheng, Zhong-Qing Huang, Jia Li, Jill Abrigo, Simon Sin-man Wong, Michael Tin-cheung Ying, Winnie Chiu-Wing Chu, Lawrence Ka-sing Wong, Thomas Wai-Hong Leung, Xiang-Yan Chen

**Affiliations:** ^1^The Russell H. Morgan Department of Radiology and Radiological Sciences, The Johns Hopkins Hospital, Baltimore, MD, United States; ^2^Department of Neurology, The Third Affiliated Hospital of Sun Yat-sen University, Guangzhou, China; ^3^Department of Medical Image Center, Yuebei People's Hospital, Shantou University Medical College, Shantou, China; ^4^Department of Health Technology and Informatics, The Hong Kong Polytechnic University, Kowloon, Hong Kong; ^5^Department of Imaging and Interventional Radiology, The Chinese University of Hong Kong, Shatin, Hong Kong; ^6^Department of Medicine and Therapeutics, The Chinese University of Hong Kong, Shatin, Hong Kong

**Keywords:** calcification, atherosclerosis, intracranial disease, computed tomography, magnetic resonance imaging

## Abstract

**Background and Purpose:** Intracranial arterial calcification (IAC) has been the focus of much attention by clinicians and researchers as an indicator of intracranial atherosclerosis, but correlations of IAC patterns (intimal or medial) with the presence of atherosclerotic plaques and plaque stability are still a matter of debate. Our study aimed to assess the associations of IAC patterns identified on computed tomography (CT) with the presence of plaque detected on vessel wall magnetic resonance imaging and plaque stability.

**Materials and Methods:** Patients with stroke or transient ischemic attack and intracranial artery stenosis were recruited. IAC was detected and localized (intima or media) on non-contrast CT images. Intracranial atherosclerotic plaques were identified using vessel wall magnetic resonance imaging and matched to corresponding CT images. Associations between IAC patterns and culprit atherosclerotic plaques were assessed by using multivariate regression.

**Results:** Seventy-five patients (mean age, 63.4 ± 11.6 years; males, 46) were included. Two hundred and twenty-one segments with IAC were identified on CT in 66 patients, including 86 (38.9%) predominantly intimal calcifications and 135 (61.1%) predominantly medial calcifications. A total of 72.0% of intimal calcifications coexisted with atherosclerotic plaques, whereas only 10.2% of medial calcifications coexisted with plaques. Intimal calcification was more commonly shown in non-culprit plaques than culprit plaques (25.9 vs. 9.4%, *P* = 0.008). The multivariate mixed logistic regression adjusted for the degree of stenosis showed that intimal calcification was significantly associated with non-culprit plaques (OR, 2.971; 95% CI, 1.036–8.517; *P* = 0.043).

**Conclusion:** Our findings suggest that intimal calcification may indicate the existence of a stable form of atherosclerotic plaque, but plaques can exist in the absence of intimal calcification especially in the middle cerebral artery.

## Introduction

Intracranial atherosclerotic disease is a leading cause of ischemic stroke worldwide, accounting for 10% of ischemic strokes in Caucasians and 30–50% in Asians (1, 2). Identification of an imaging marker of vulnerable plaques that are prone to rupture and lead to downstream thromboembolic events is of vital importance in the early detection of high-risk patients (3). Intracranial artery calcification (IAC) is a highly prevalent finding on non-contrast enhanced head CT scans in both stroke patients (4) and the general population (5). Several epidemiological studies have shown that IAC is an independent risk factor of future ischemic cerebrovascular events (5, 6). Although IAC seen on CT is traditionally thought as a surrogate marker for atherosclerosis, the association between IAC and plaque stability is controversial.

Calcification is located not only in the tunica intima (intimal calcification) as typically seen with atherosclerotic plaque but also in the tunica media or around the internal elastic lamina (medial calcification), which is not a typical feature of atherosclerotic plaque (7). Previous studies have found that intimal and medial calcification show differences in the neurovascular risk factor profile, with intimal calcification being related to smoking and hypertension whereas medial calcification more associated with diabetes, older age, and chronic kidney disease (8–10). This indicates that the two distinct morphological patterns of IAC may represent different pathological processes that have distinct clinical outcomes including cerebrovascular ischemic events (11). Our recent pathological study examining large intracranial arteries showed that intimal calcification existed in advanced atherosclerotic lesions and could be used as a marker for intracranial atherosclerosis, whereas medial calcification was not associated with atherosclerosis (12). Despite the histologic evidence, it remains unclear in clinical practice if intimal calcification should be considered as a feature of plaque stability.

High-resolution vessel wall magnetic resonance imaging (VWMRI) has gained notoriety as an imaging approach to reliably visualize the intracranial vessel wall, leading to the non-invasive characterization of intracranial atherosclerotic plaques (13–15). While VWMRI can detect soft plaque components (e.g., intraplaque hemorrhage, lipid core) (16–18), CT is superior for characterizing calcification (19, 20). Therefore, in this study, we aimed to identify the patterns of IAC on CT to determine their associations with the presence of plaque detected by VWMRI and plaque stability.

## Materials and Methods

### Subjects

Patients admitted to the stroke center at the Prince of Wales Hospital from 2014 to 2018 were recruited for the VWMRI exams, with the following inclusion criteria: (1) ischemic stroke confirmed by MRI or clinical evidence of TIA within 30 days; (2) Moderate or severe luminal stenosis in at least one intracranial artery as confirmed by magnetic resonance angiography; (3) one or more atherosclerotic risk factors, including hypertension, diabetes mellitus, hyperlipidemia, and smoking. The exclusion criteria for the VWMRI exams were as follows: (1) contraindications to MRI; (2) non-atherosclerotic vasculopathies, such as arteritis, dissection, or Moyamoya disease; (3) evidence of cardioembolism, such as atrial fibrillation. Patients who had good VWMRI images and underwent routine CT were recruited in this study. The study was approved by the Clinical Research Ethics Committee of the Chinese University of Hong Kong.

### CT Acquisition and Processing

Routine CT was performed at the admission on a 64-slice multi-detector row CT system (Light speed 64 plus, General Electric, Milwaukee, WI, USA) without contrast administered. All unenhanced brain CT scans were acquired in axial mode with tilting along the occipito-meatal line, covering the base of the skull to the vertex region. Imaging acquisition parameters were as follows: slice thickness 5 mm, 120 kVp, 170 mAs, 1 s per rotation. Axial images were reconstructed at 0.625-mm intervals.

**Figure 1:**
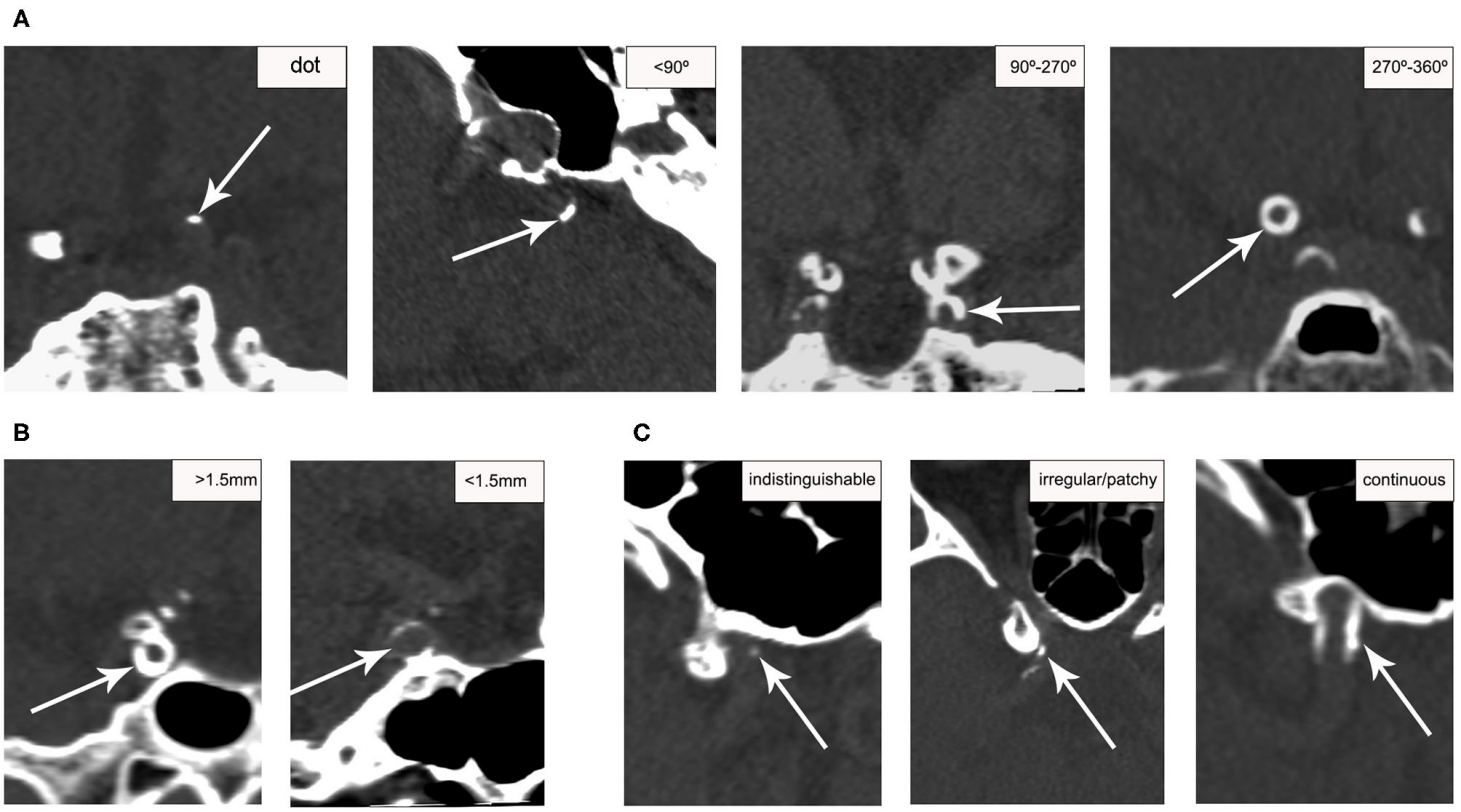
Examples of calcification score for categorizing ICA calcification patterns. Calcification (arrows) was scored based on circularity **(A)**, thickness **(B)**, and continuity **(C)** on CT. Circularity and thickness were assessed on the short axis; continuity was assessed on the long axis.

The presence and morphologic characteristics of IAC were assessed for the major cerebral arteries: cavernous segment (C3) of the intracranial carotid artery (ICA), supraclinoid segment (C4) of the ICA, M1 segment of the middle cerebral artery (MCA), the basilar artery (BA), and the intracranial segment (V4) of the vertebral artery (VA). For all patients, the assessment was done on the reconstructed three dimensional (3D) CT images by one of two readers (WJ. Y. and ZQ. H.) with at least 2 years of experience reading CT images, who were blinded to the clinical data and MRI results. The presence of IAC was defined as hyperdense foci with a density of more than 130 Hounsfield units (HU). The patterns of calcification were categorized based on a previously established calcification scoring model (21), in which scores were assigned for morphologic patterns of calcification as follows: circularity (1 for dots, 2 for <90°, 3 for 90–270°, and 4 for 270–360°), thickness (1 for thick ≥1.5 mm, and 3 for thin <1.5 mm), and continuity of calcification over the long axis of arterial segments (0 for indistinguishable, 1 for irregular/patchy, and 4 for continuous) (Figure 1). The cumulative calcification score was used to localize the calcification on a spectrum from intimal to medial, with predominantly intimal ranging from 1 to 6 points and predominantly medial ranging from 7 to 11 points. To assess the inter-reader reliability, two raters (WJ. Y. and ZQ. H.) reviewed 20 randomly selected CT images independently, and graded all calcifications using the above scoring system.

### MRI Acquisition and Processing

VWMRI was performed using a 3T Achieva MR system (Philips Healthcare, Cleveland, OH, USA) with an 8-channel head coil. The protocol included a transverse 3D T1-weighted Volumetric ISotropic Turbo spin-echo Acquisition (VISTA) sequence and a time-of-flight MR angiography (MRA) sequence, as described before (22, 23). The following scan parameters were used for the T1-weighted VISTA: field-of-view 200 × 167 × 45 mm^3^, acquired resolution 0.6 × 0.6 × 1.0 mm^3^, reconstructed resolution 0.5 × 0.5 × 0.5 mm^3^, TR 1,500 ms, TE 36 ms, SENSE factor 1.5 (phase-encode direction), echo spacing 4.0 ms, TSE + startup echoes 56 + 6. VWMRI images were repeated 20 min after the intravenous injection of 0.2 mL/kg of a gadolinium-containing gadolinium-containing contrast agent (Dotarem; Guerbet, Roissy CdG Cedex, France) at an injection rate of 3.5 ml/s. Scan parameters for the time-of-flight MRA sequence were as follows: FOV 200 × 200 × 56 mm^3^, acquired resolution 0.4 × 0.6 × 0.7 mm^3^, TR/TE 23/3.5 ms.

VWMRI was acquired within 30 days after the onset of ischemic events. The presence of atherosclerotic plaque was identified independently by 2 analysts (WJ. Y. and L. Z.) with more than 3 years of experience in VWMRI who were blinded to the clinical data and CT results. Discrepancies were resolved by consensus with a third analyst (XY. C.). The 3D VW images were reconstructed orthogonal to the vessel axis at 0.5-mm intervals. An atherosclerotic plaque was defined as wall thickening identified on the short axis on both pre- and post-contrast VWMRI. Each detected plaque was classified as a culprit, non-culprit, or indeterminate lesion according to its likelihood of causing the downstream ischemic cerebrovascular events, as previously described (24). A culprit lesion was defined if it was the only lesion within the vascular territory relative to the stroke. If there were multiple plaques in the same affected vascular territory, the most stenotic plaque was classified as a culprit lesion, and the others as indeterminate lesions. A non-culprit lesion was defined as one that was not within the vascular territory of the stroke. The degree of stenosis for each atherosclerotic plaque was measured on maximum intensity projection images reformatted from time-of-flight MRA, following methods established by the Warfarin-Aspirin Symptomatic Intracranial Disease (WASID) trial (25): percent stenosis = (1- D_stenosis_/D_normal_) ^*^100.

### Matching Calcification With Atherosclerotic Plaques

After the identification of plaques on VWMRI and calcification patterns (intimal or medial) on CT, a side-by-side analysis was performed, in which the rater was shown the co-registered CT and MRI images to check if the identified IAC corresponds to any detected plaques.

### Statistical Analysis

Continuous quantitative variables are described as mean ± SD and a percentage is used to describe the categorical variables. The data were analyzed using the SPSS 20.0 software package (SPSS, Inc., USA). Fisher's exact test was used for comparing the prevalence of calcification between culprit and non-culprit lesions. Multivariate logistic regression (adjusted for the degree of stenosis) was used to assess the association between calcification and culprit plaques, with mixed models to account for repeated measurements within subjects. Inter-reader agreements were estimated using Cohen κ. Reliabilities below 0.4 were characterized as poor, 0.4–0.75 as fair to good, and above 0.75 were considered as excellent. *P* < 0.05 was considered to indicate a statistically significant difference.

## Results

In total, 75 patients (mean age, 63.4 ± 11.6; male, 46) were included in this study. The clinical characteristics of the study population are shown in Table 1.

**Table 1:** Clinical characteristics of the study population (*n* = 75).

Age	63.4 ± 11.6
Male	46 (61.3%)
Hypertension	52 (69.3%)
Diabetes	19 (25.3%)
Hyperlipidemia	37 (49.3%)
Smoking	
Current	7 (9.3%)
Ex-smoker	20 (26.7%)
Ischemic stroke	67 (89.3%)
Transient ischemic attack	8 (10.7%)

*Values are presented as mean ± SD or number (%)*.

### Distribution of Calcification on CT and Plaques on VWMRI

A total of 637 segments were examined (on average 8.5 segments per patient), out of which 221 (34.7%) segments with calcification were identified on CT from 66 (88.0%) subjects. Of the 66 subjects showing calcification, most had 1 to 4 segments with calcification (21.2, 21.2, 15.2, 16.7%, respectively), and few patients (3.0%) had as many as 8 segments with calcification. Calcification predominantly occurred in the cavernous (41.6%) and supraclinoid segments (35.3%) of the ICA, followed by the V4 segment (19.9%), BA (1.8%), and MCA (1.4%) (Table 2).

**Table 2:** Distributions of calcification assessed on CT and atherosclerotic plaques assessed on VWMRI in the segments of large intracranial arteries.

	**Calcification**			**Plaques**
	**Intimal**	**Medial**	**Total**	
ICA				
C3	24 (27.9%)	68 (50.4%)	92 (41.6%)	19 (6.7%)
C4	30 (34.9%)	48 (35.6%)	78 (35.3%)	67 (23.6%)
M1	3 (3.5%)	0 (0.0%)	3 (1.4%)	110 (38.7%)
V4	26 (30.2%)	18 (13.3%)	44 (19.9%)	47 (16.5%)
BA	3 (3.5%)	1 (0.7%)	4 (1.8%)	41 (14.4%)
Total	86	135	221	284

*ICA, internal carotid artery; C3, cavernous segment; C4, supraclinoid segment; M1, M1 segment of the middle cerebral artery; V4, V4 segment of the vertebral artery; BA, basilar artery*.

**Figure 2:**
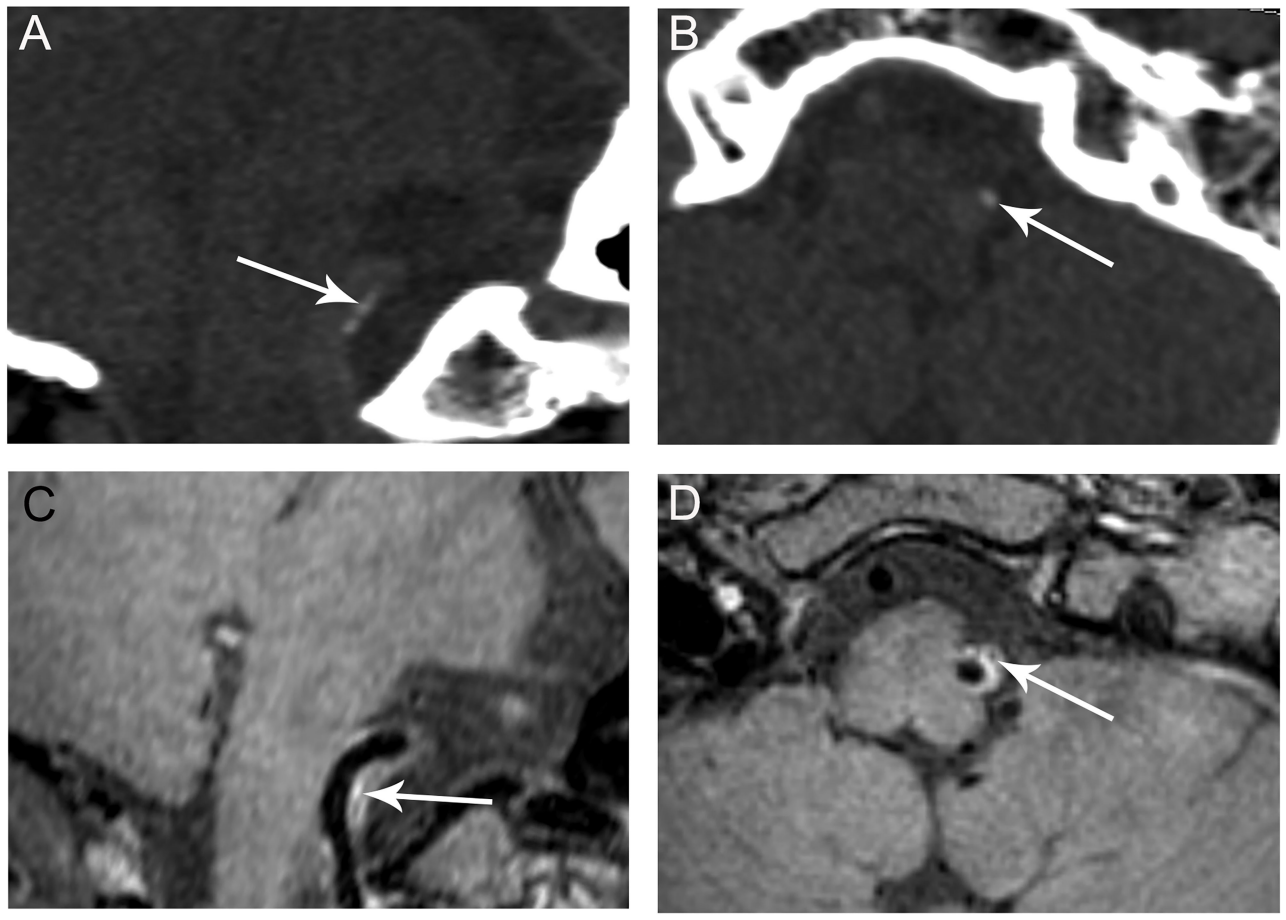
Representative images of predominant intimal VA calcification. CT shows a small clustered calcification on the long (**A**, arrow) and short axes (**B**, arrow) of the right VA indictive of intimal calcification. The calcification corresponds to a hypointensity area (**C,D**, arrows) within an atherosclerotic plaque observed on VWMRI.

**Figure 3:**
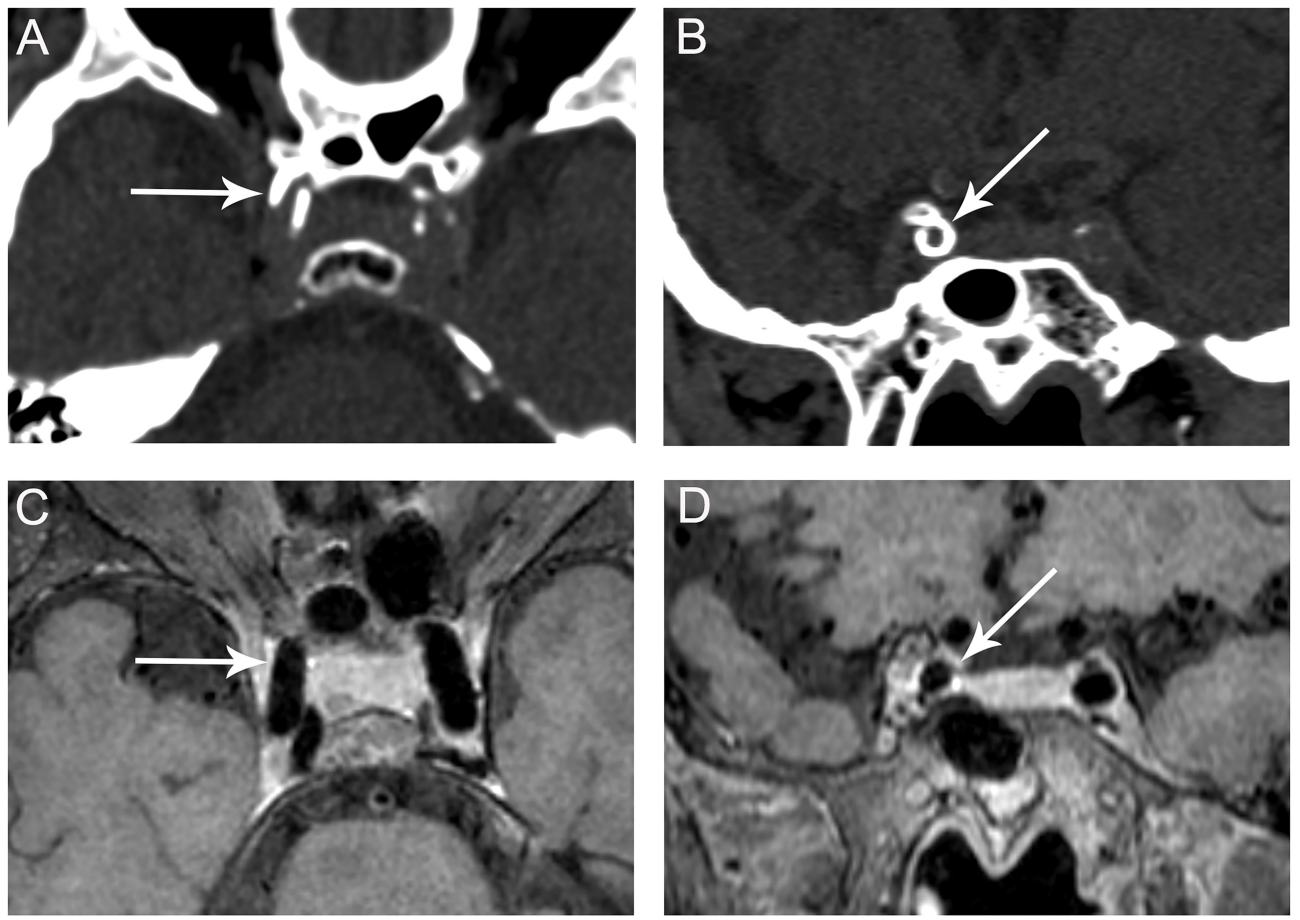
Representative images of predominant medial ICA calcification. CT shows a continuous, circumferential calcification on the long (**A**, arrow) and short axes (**B**, arrow) of the right cavernous segment of the ICA suggesting medial calcification. No corresponding plaque is observed on VWMRI (**C**, **D**, arrows).

Among the 221 segments with calcification, 86 (38.9%) calcifications were categorized as predominantly intimal calcification (Figure 2, Supplementary Figures 1, 2) and the other 135 (61.1%) as predominantly medial calcification (Figure 3). The majority of calcifications involving the cavernous and supraclinoid segments were medial calcification [68/92 (73.9%) and 48/78 (61.5%), respectively]. In comparison, among V4 segments with calcification, 18 out of 44 (40.9%) showed medial calcification.

There were a total of 284 plaques detected on VWMRI in all participants, most commonly involving the M1 segment (38.7%), followed by the cavernous and supraclinoid segments of the ICA (30.3%), the V4 segment (16.5%), and then BA (14.4%) (Table 2).

### Correlation Between Calcification Patterns and Plaques

Since the distal V4 segment was not imaged by VWMRI for 31 patients, there was no corresponding VWMRI for 18 VA segments harboring calcification, including 11 intimal calcifications and 7 medial calcifications. In the remaining 203 segments with calcification, 75 (36.9%) segments showed intimal patterns and 128 (63.1%) showed medial patterns of calcification. Of the 75 intimal calcifications, 54 (72.0%) were co-existent with atherosclerotic plaques. In comparison, only a minority of medial calcifications (13/128, 10.2%) corresponded with atherosclerotic plaques (Table 3). Among the 67 atherosclerotic plaques with calcification (54 intimal and 13 medial), only 6 (9.0%) were culprit, 49 (73.1%) were non-culprit, and 12 were indeterminate lesions (17.9%).

**Table 3:** Correlation between calcification patterns on CT and the presence of plaques on VWMRI.

		**Presence of plaques on VWMRI**		
		**Yes**	**No**	**Total**
Patterns of calcification on CT	Intimal	54 (72.0%)	21 (28.0%)	75
	Medial	13 (10.2%)	115 (89.8%)	128
	Total	67 (33.0%)	136 (67.0%)	203

Sixty-four (22.5%) out of the 284 detected plaques were culprit, 189 (66.5%) were non-culprit, and 31 (10.9%) were indeterminate lesions. There was a higher prevalence of calcification in non-culprit lesions than in culprit lesions (25.9 vs. 9.4%, *P* = 0.008). When we categorized calcification by intimal and medial calcification, 5 (7.8%) plaques with intimal calcification were culprit lesions and 39 (20.6%) plaques with intimal calcifications were non-culprit (*P* = 0.021). Only 1 (1.6%) medial calcification was found in culprit lesions and 10 (5.3%) were observed in non-culprit lesions. No significant difference in the prevalence of medial calcification was found between culprit and non-culprit plaques (*P* = 0.299) (Table 4). Culprit plaques showed significantly higher degree of stenosis than non-culprit plaques in lesions with (64.3 ± 28.1 vs. 30.6 ± 24.9, *P* = 0.003) and without calcification (65.6 ± 32.5 vs. 28.4 ± 23.8, *P* < 0.001).

**Table 4:** Association between calcification patterns and culprit plaques.

	**Culprit (*n* = 64)**	**Non-culprit (*n* = 189)**	*P*-value^*^
All calcification	6 (9.4%)	49 (25.9%)	0.008
Intimal calcification	5 (7.8%)	39 (20.6%)	0.021
Medial calcification	1 (1.6%)	10 (5.3%)	0.299

*%, percentage of all calcification, intimal calcification, and medial calcification in culprit (n = 64) and non-culprit (n = 189) plaques, respectively*.

* *P-values were generated by using Fisher's exact test*.

The mixed logistic regression model adjusting for the degree of stenosis showed that calcification was associated with non-culprit plaques [odds ratio (OR), 3.068; 95% confidence interval (CI), 1.171–8.037; *P* = 0.023]. After categorizing calcification to intimal and medial patterns, intimal calcification was associated with non-culprit plaques (OR, 2.971; 95% CI, 1.036–8.517; *P* = 0.043), whereas medial calcification was not associated with non-culprit plaques (OR, 2.423; 95% CI, 0.303–19.354; *P* = 0.606).

### Reliability Assessment

Inter-reader agreement for the presence of calcification was excellent (κ: 0.911). Inter-reader agreement for classifying calcification was excellent (κ: 0.803). Inter-reader agreement for the presence of plaques was also excellent (κ: 0.735).

## Discussion

In this study, we found that IAC was common in patients with ischemic cerebrovascular disease, and the ICA was most frequently affected. Continuous, thin, and circular IAC detected on CT indicative of medial calcification was less likely to be co-existent with atherosclerotic plaques. In contrast, discontinuous, thick, and focal IAC that suggests intimal calcification was mostly observed in plaques and might be a feature of plaque stability.

A previous CT study has reported that ICA was the most common site affected by calcification (60%) in Chinese patients, followed by the VA (20%), MCA (5%), and BA (5%) (26). We found 77% calcification involving the cavernous segment and supraclinoid segments of the ICA in the present study, which is higher than the 60% reported in the literature because the cavernous segment and supraclinoid segment were calculated separately. Despite the high prevalence of plaques (38.7%) involving the MCA in our study, calcification was not common (1.4%) in the MCA, which indicates that the absence of CT calcification in MCA territory cannot exclude the presence of MCA plaques. A CT study of 1,132 stroke patients showed the presence of dominant intimal calcification in 30.9% of participants and dominant medial calcification in 46.9% (8). Using the same grading system, we found that medial calcification accounted for 60% of all calcifications. Medial calcification was especially high in the intracranial ICA (68.2%), concordant with the histopathological finding that 71% of ICA calcifications are medial (27). Medial calcification was only observed in ICA and VA, suggesting that specific vascular beds might behave differently in terms of the pathogenesis of plaque and related vascular events (28).

Consistent with our prior histopathological study (12), the present clinical study demonstrated that most intimal calcification was observed in atherosclerotic plaques, whereas the majority of medial calcification was not related to plaques. Intimal calcifications were more commonly found in non-culprit lesions than in culprit lesions. Ischemic cerebrovascular events in patients with intracranial atherosclerosis are frequently caused by rupture of vulnerable plaque and the subsequent thromboembolism (29). Therefore, the more frequent intimal calcification in non-culprit lesions may indicate the stabilized effect of IAC on intracranial atherosclerosis.

In extracranial arteries, a calcified plaque is considered more biomechanically stable than a non-calcified plaque (30). Huang et al. (31) showed that calcification did not increase fibrous cap stress in either ruptured or stable coronary atherosclerotic plaques, and thus did not contribute to plaque rupture and the subsequent thrombotic events. Another carotid plaque study found the extent of plaque calcification was inversely related to plaque fibrous cap inflammation and symptomatic plaques were less calcified (32). However, the effect of calcification on plaque stability remains controversial in intracranial arteries. A recent clinical study of patients with acute stroke in the MCA territory showed that the presence of calcification in the MCA was less frequent in the symptomatic group, which suggests that calcification might have a protective effect on symptomatic MCA infarction (33). Nonetheless, the symptomatic lesion in their study was defined as a stenotic MCA detected on MRA upstream from an infarction, but angiographic imaging may underestimate the stenosis and severity of atherosclerosis (34), highlighting the value of plaque detection using VWMRI. In the present study, by matching calcification detected on CT with atherosclerotic plaque seen on VWMRI, we showed that intimal calcification was associated with non-culprit lesions, independent of the degree of stenosis, indicating that intimal calcification may help to stabilize atherosclerotic plaques. In contrast to our results, some prior studies suggested that plaque calcification may predict stroke risk (4, 35). The discrepancies may be explained by the hypothesis that the presence of IAC seen on CT may only denote a cumulative imaging marker of atherosclerosis burden (36), but IAC itself is not a source of embolism or thrombosis (37–39).

Of note, some segments with intimal calcification did not show any corresponding atherosclerotic plaques. In this study, medial calcification was defined as circumferential, thin, and continuous whereas intimal calcification was clustered, thick, and scattered. However, according to prior pathological studies, medial calcification progresses along the internal elastic lamina from a scattered spotty pattern in its early stage to a confluent circumferential pattern in its late-stage (7). Therefore, some early granular medial calcifications could be misclassified as intimal in the present study. Moreover, Vos et al. (27) reported that a small proportion of intimal calcification occurred in early atherosclerotic lesions (intimal thickening and intimal xanthoma) that could appear as normal or minimal wall thickening on VWMRI. Given that CT is more sensitive to calcification than VWMRI to plaques, it is very likely that intimal calcifications were detected on CT while the corresponding plaques were too small to resolve on VWMRI. VWMRI was performed at a field strength of 3T, which is needed to accurately differentiate pathological vessel wall changes from a normal arterial wall compared with VWMRI at 1.5 T that is more common in regional and community hospitals (40). Some small plaques identified on 3 T MRI may become undetectable on 1.5 T scanner, so we would expect a higher frequency of intimal calcification without corresponding plaques detected on 1.5 T VWMRI.

There are several limitations to our study. First, since the calcification types were classified based on a prior CT scoring system that was developed and validated in the ICA calcification in only 16 patients, caution should be taken when extrapolating these findings to other intracranial arteries. We have validated the scoring system in the basilar and vertebral arteries in a prior histology study (12). Although only 11 calcifications (9 intimal and 2 medial calcifications) were included in that study, all of them were accurately classified based on this CT score, indicating that the scoring system can be generalized to the vertebrobasilar system. Further studies with a larger sample size are warranted to test the reliability of the scoring system in categorizing calcification involving other intracranial arteries. Second, the prevalence of intracranial plaques was underestimated because the V4 segment was not covered by VWMRI in 31 patients. Third, we qualitatively assessed the presence or absence of calcification but did not quantify the volume and assess the morphology of calcification. Priors studies have demonstrated that compared with the deep, coalescent calcification, superficial and scattered calcification was more associated with plaque instability (41, 42). Furthermore, some scattered micro-calcifications that may be more related to culprit plaques (43) are beyond the resolution of a CT scan (44), which may lead to an underestimation of the prevalence of calcification in plaques. Lastly, since IAC is more prevalent in Asians, African Americans, and Hispanics than in Caucasians (20, 26), larger studies involving other populations are needed to increase the generalizability of these findings.

## Conclusions

In summary, our study indicates that intimal calcification, unlike medial calcification, is a CT maker of atherosclerotic plaque, but a lack of intimal calcification on CT does not exclude its presence, especially in the MCA. Intimal calcification is more associated with non-culprit plaques and may be a potential marker of plaque stability. Our study results may provide basis for the management of stroke patients with calcification.

## Data Availability Statement

The original contributions presented in the study are included in the article/Supplementary Material, further inquiries can be directed to the corresponding author/s.

## Ethics Statement

The studies involving human participants were reviewed and approved by The Institutional Review Board of the Chinese University of Hong Kong. The patients/participants provided their written informed consent to participate in this study.

## Author Contributions

W-JY recruited patients, analyzed data, and drafted the manuscript. BW participated in the study design and revised the manuscript. LZ recruited patients and analyzed imaging data. Z-QH and JL analyzed the data. JA, SW, and MY acquired the VWMRI data. WC, LW, and TL participated in the design and coordination of the study. X-YC conceived the study, participated in its design and coordination, and revised the manuscript. All authors contributed to the article and approved the submitted version.

## Conflict of Interest

The authors declare that the research was conducted in the absence of any commercial or financial relationships that could be construed as a potential conflict of interest.
