# Supplementary material for: Understanding the Clinical Implications of Intracranial Arterial Calcification Using Brain CT and Vessel Wall Imaging
Source: Front Neurol. 2021 Jul 15;12:619233. doi: 10.3389/fneur.2021.619233 (PMC8319500; doi:10.3389/fneur.2021.619233)
Supplement: Supplementary file 1 [file Data_Sheet_1.docx]

Supplementary Material

**
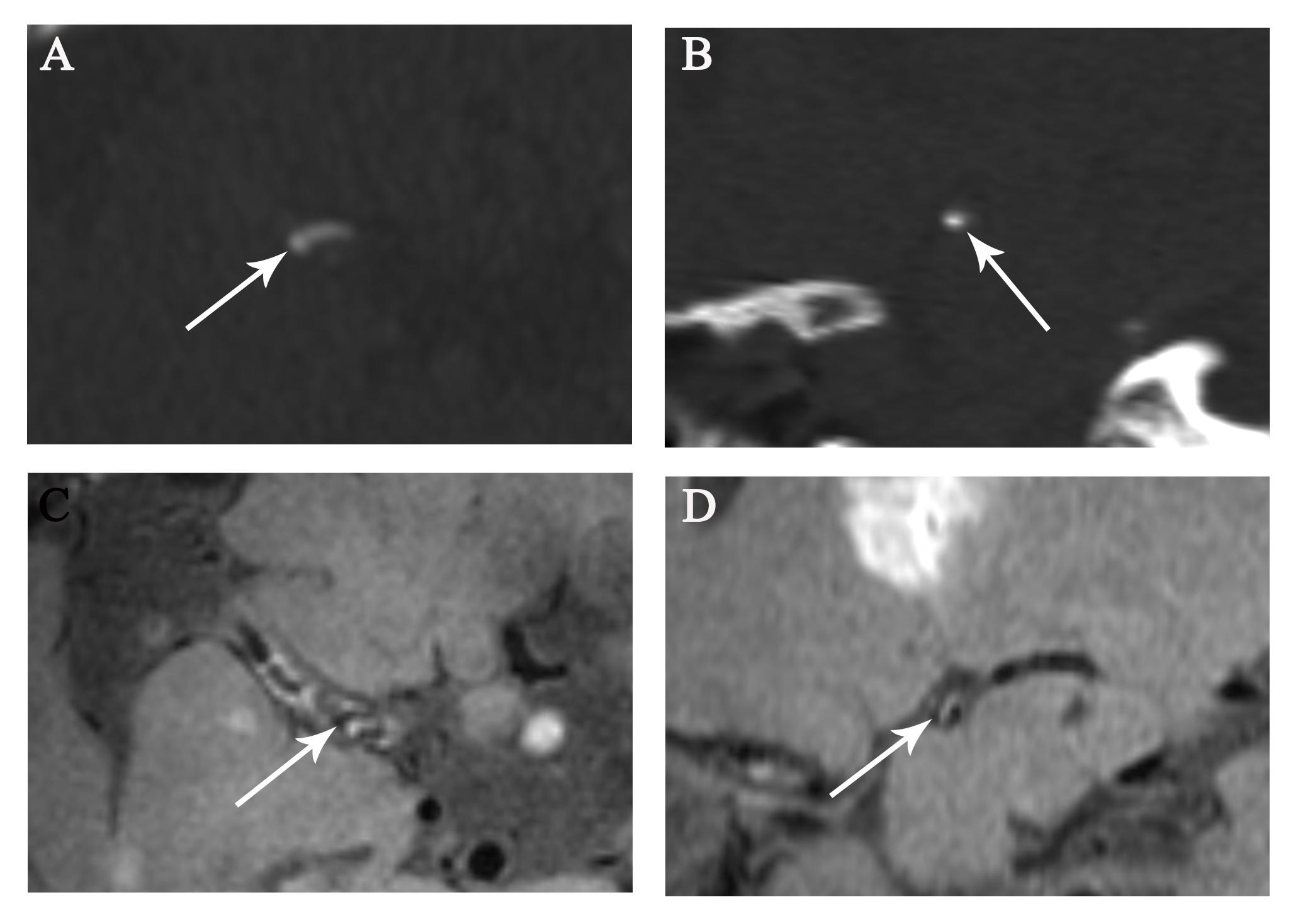
**

**Supplementary Figure 1.** Representative images of predominant intimal MCA calcification. CT shows a large clustered calcification on the long (**A,** arrow) and short axes (**B**, arrow) of the right MCA suggestive of intimal calcification, which corresponds to a hypointensity area (**C** and **D**, arrows) within a large atherosclerotic plaque on VWMRI.

**
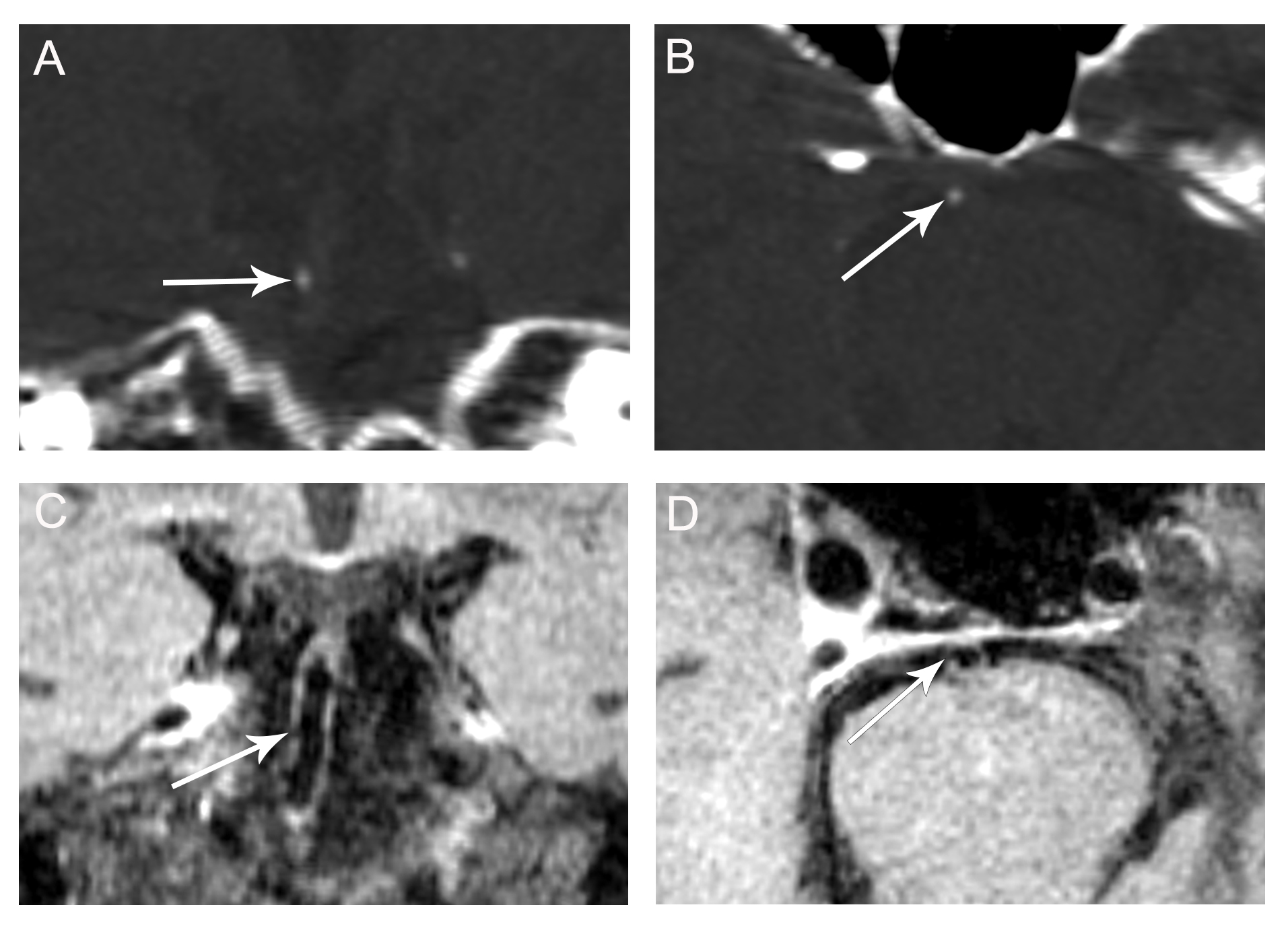
**

**Supplementary Figure 2.** Representative images of predominant intimal BA calcification. CT shows a small clustered calcification on the long (**A,** arrow) and short axes (**B**, arrow) of the BA suggestive of intimal calcification, which corresponds to a hypointensity area (**C** and **D**, arrows) in an atherosclerotic plaque seen on VWMRI.
